# Epigenetic Mechanisms of Obesity: Insights from Transgenic Animal Models

**DOI:** 10.3390/life15040653

**Published:** 2025-04-16

**Authors:** Elisa S. Na

**Affiliations:** School of Social Work, Psychology, & Philosophy, Texas Woman’s University, Denton, TX 76209, USA; ena@twu.edu; Tel.: +1-940-898-2307

**Keywords:** epigenetics, obesity, allostasis, hypothalamus, ultra-processed foods

## Abstract

Obesity is a chronic disease with prevalence rates that have risen dramatically over the past four decades. This increase is not due to changes in the human genome but rather to environmental factors that promote maladaptive physiological responses. Emerging evidence suggests that external influences, such as high-fat diets, modify the epigenome—the interface between genes and the environment—leading to persistent alterations in energy homeostasis. This review explores the role of epigenetic mechanisms in obesity, emphasizing insights from transgenic animal models and clinical studies. Additionally, we discuss the evolution of obesity research from homeostatic to allostatic frameworks, highlighting key neuroendocrine regulators of energy balance.

## 1. Introduction

Obesity rates have tripled in the last 40 years with an expected 1 billion of the world’s population predicted to meet the criteria of obesity by 2025 [1]. Currently, 40% of American adults are classified as obese, with worldwide obesity rates estimated to be at 12% [1]. Particularly noteworthy is that there is a strong correlation between childhood obesity and adulthood obesity with simulation models predicting that 59% of 2-year-olds will maintain obese phenotypes by the age of 35 [2].

Obesity is defined as a body mass index (BMI) equal to or above 30 with the ratio of weight (kg) and height (m) being the determining factors. This definition of obesity has fallen out of favor, however, and has undergone significant revision with concurrent measures of obesity now being required for its diagnosis. Measurements such as waist circumference, visceral fat, and body adiposity index, among others, are being used in conjunction with BMI to diagnose obesity [3].

Obesity is a significant risk factor for a number of different maladies: cancer, heart disease, stroke, osteoarthritis, and pulmonary diseases being chief among them [4]. Mental health disorders such as depression and anxiety are also highly co-morbid with obesity [5], illustrating the pervasive effects of obesity not just on physiological health but on mental health as well. Given the many adverse consequences of obesity, it has become clear that strategies to mitigate this public health crisis should take precedence as they could also lead to the amelioration of these co-morbid diseases. However, despite its strong association with these disease states, the incidence of obesity continues to climb at an alarming rate so much so that the Centers for Disease Control (CDC) and the World Health Organization (WHO) now consider obesity to be an epidemic [6].

**Figure 1:**
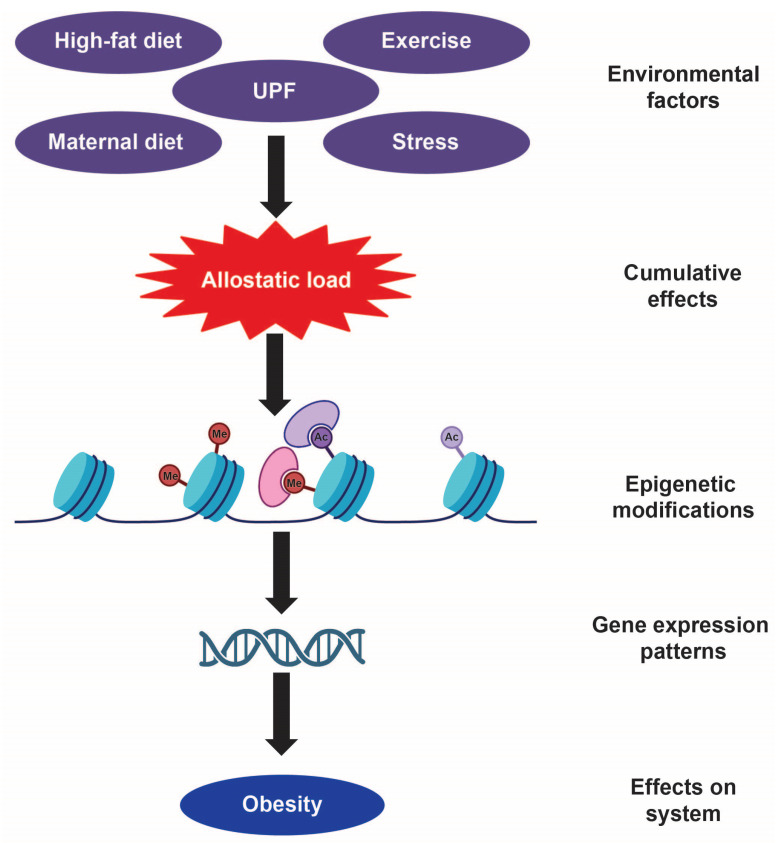
The effects of environmental factors on the epigenome. External variables such as ultra-processed foods (UPFs), stress, maternal diet, exercise habits, and high-fat diet increase allostatic load to the system, causing changes to the epigenome. This, in turn, changes gene expression patterns which lead to maladaptive states such as obesity.

The origins of obesity have become difficult to disentangle largely due to the multiple factors that are likely contributors to its etiology. Genetic predisposition, dietary and exercise habits, aging, and even a person’s intrauterine environment have been proposed to have compounding effects on obese pathology [7]. While genes have been the primary focus of investigation/research efforts, previous work has demonstrated that individuals who carry “obese genes” do not necessarily become overweight as healthy lifestyles can counteract the effects of these genes [8]. Moreover, the human genome has remained relatively stable over the last 40 years [8] with genetic variants accounting for only a small fraction of obese cases. Thus, other underlying factors must be responsible for its pathophysiology. It has been hypothesized that modifications to our epigenome, a collection of heritable changes to our genes that alter their expression, are one of the primary driving factors of obesity. The epigenome is particularly permissive to environmental cues such as sedentary lifestyles, diet, exposure to ultra-processed foods, maternal nutrition, and aging among many other factors (Figure 1). This review will describe the evolution of the theoretical framework of obesity from its ideological origins of homeostasis to its current conceptualization of being an allostatically regulated process. This review will also provide a brief overview of central mechanisms governing energy balance, focusing on the hypothalamus and its related neuropeptides. Lastly, using clinical cases as well as transgenic animal models of obesity, this review will discuss the interaction between obesogenic diets, the epigenome, as well as ensuing transcriptional changes that are at the heart/crux of the obesity epidemic.

## 2. Ideological Underpinnings of Obesity

Many have considered obesity to be a disease of body energy dysregulation. Obesity is not an advantageous state for any physiological system as excess stored energy in the form of fat depots would increase fatigue, reduce mobility, and potentially impair muscle performance [9]. How is it that an obese body maintains this maladaptive state and in some instances will defend this condition against perturbations such as a caloric deficit? The phenomenon of weight rebound is one such example in which weight is rapidly regained after medical/surgical interventions [10,11,12]. These clinical observations suggest the difficulty in trying to maintain permanent weight loss and indicate that other factors must be at play to undermine weight loss mitigation efforts.

In order to grasp the complexity of obesity, it is important to understand its ideological origins. In general, obesity may arise as a result of an excess of energy input and a deficiency in energy output. Energy homeostasis is the body’s way of regulating its energy intake relative to its energy output and there are many systems in place to ensure its optimal functioning. Historically, the term homeostasis derives its meaning from the French physiologist, Claude Bernard, who coined the term milieu interièur to account for the body’s “constancy of [its] internal environment… [the] equilibrium [of which] results from continuous and delicate compensation established as if the most sensitive of balances” [13]. This concept later evolved to become homeostasis, so named by the American physiologist Walter B. Cannon who maintained that [14] disturbances to one’s physiology are “normally kept within narrow limits …wide oscillations are prevented and the internal conditions are held fairly constant”. These hypotheses set forth by Bernard and Cannon provided a framework for our current conceptualization of homeostasis but suggest that set points are predetermined in such a way as to provide relatively little flexibility in physiological systems. This, in essence, limits the responsivity of our systems to environmental influences. If it is true that our physiologies are maintained within these “narrow limits”, then incidences of obesity would exist as more of a statistical outlier. Given the status of our current obesity crisis, the idea of homeostasis is no longer relevant and must be revised to account for the alarming rise in obesity cases.

**Figure 2:**
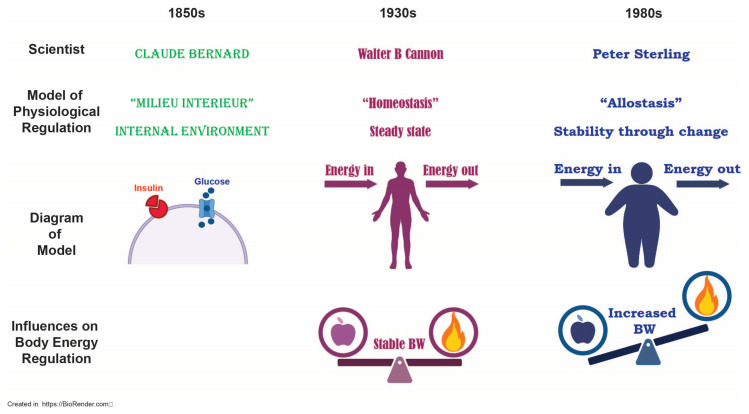
Evolution of the concept of allostasis.

Sterling, a neuroscientist and physiologist, countered that the term homeostasis by definition was an “inherently inefficient” process and introduced the concept of allostasis, whereby an organism is able to anticipate needs in order to prepare biological systems to meet them “before they arise” [15]. According to Sterling, the brain is continuously updating its system based on environmental information to prevent aberrant need states. This particular theory may be a more parsimonious explanation for maladaptive conditions such as obesity, in which the obese state is considered to be a “modern disorder of regulation”. Obesity becomes a positive feedback mechanism; an “increased demand” drives an increase in “response capacity” [15]. This shift in response capacity changes the organism in such a way that reversing course (i.e., decreasing caloric intake) fails to alter the range to which the organism has become accustomed. This, in essence, drives the “need” state past the point of what is calorically required and instead creates a “need” state driven to excess (Figure 2).

Sterling’s allostatic hypothesis revolutionized our current understanding of maladaptive physiological processes such as obesity, suggesting that our shifting response capacities are to blame. Sterling posits that obesity is the result of a loss in regulatory capacity, whereby the organism can no longer reflexively respond to ever-shifting energy demands [15]. Thus, dysregulation of allostatic processes could undermine weight loss efforts, as chronic caloric restriction would be unable to counter allostatic dysfunction. While this hypothesis provides an alternative conceptual explanation for obesity, it does not necessarily identify its etiological underpinnings. As mentioned previously, the pathophysiology of obesity is the culmination of genetic and environmental risk factors and, as such, the mitigation of this disease is an incredibly complex and nuanced issue. Experience with obesogenic diets fundamentally changes allostatic processes, which suggests that genetic factors are unlikely to be the cause of the majority of obesity cases. Thus, one could argue that excessive calorie intake might “stress” physiological systems to deal with the increased energy surplus which would result in the activation of allostatic mechanisms. Over time and with chronic exposure to calorie surfeit, pronounced changes to allostatic load [16] will ensue, to the point where one cannot “roll back the clock” [16,17]. Chronic excessive food intake, in addition to other factors such as sedentary lifestyles, and stress have long-term consequences and, as such, these cumulative experiences fundamentally challenge the biological constraints that regulate body energy homeostasis. The accumulation of these life events produces “persistent molecular signatures…with long-lasting genomic effects” [18] in the form of changes to our epigenomes, the molecular interface between environmental factors and gene expression. Our epigenomes respond reflexively to modern Westernized diets as allostatic responses. The responses become maladaptive and produce a perseverative allostatic load whereby the organism can no longer roll back the metaphorical clock. This increased allostatic load may be one explanation for why obesity is so challenging to overcome, as epigenetic mechanisms, once altered by the experience of obesogenic diets, become relatively fixed.

## 3. Central Mechanisms Governing Body Energy Homeostasis

In order to understand how metabolism is influenced by allostatic loads it is important to discuss neurobiological mechanisms that govern body energy homeostasis, the most important of which is the master gland, the hypothalamus. The hypothalamus is a small but critical structure in the brain. Given that it occupies only 1% of our total brain weight (4 g out of the 1.2 to 1.4 kg total brain weight), this caudal structure carries great importance in the context of our physiology [19]. Its functions are wide-ranging and include body fluid and energy homeostasis, reproductive behaviors, thermoregulation, the regulation of circadian rhythms, sleep, and emotional expression [19].

**Figure 3:**
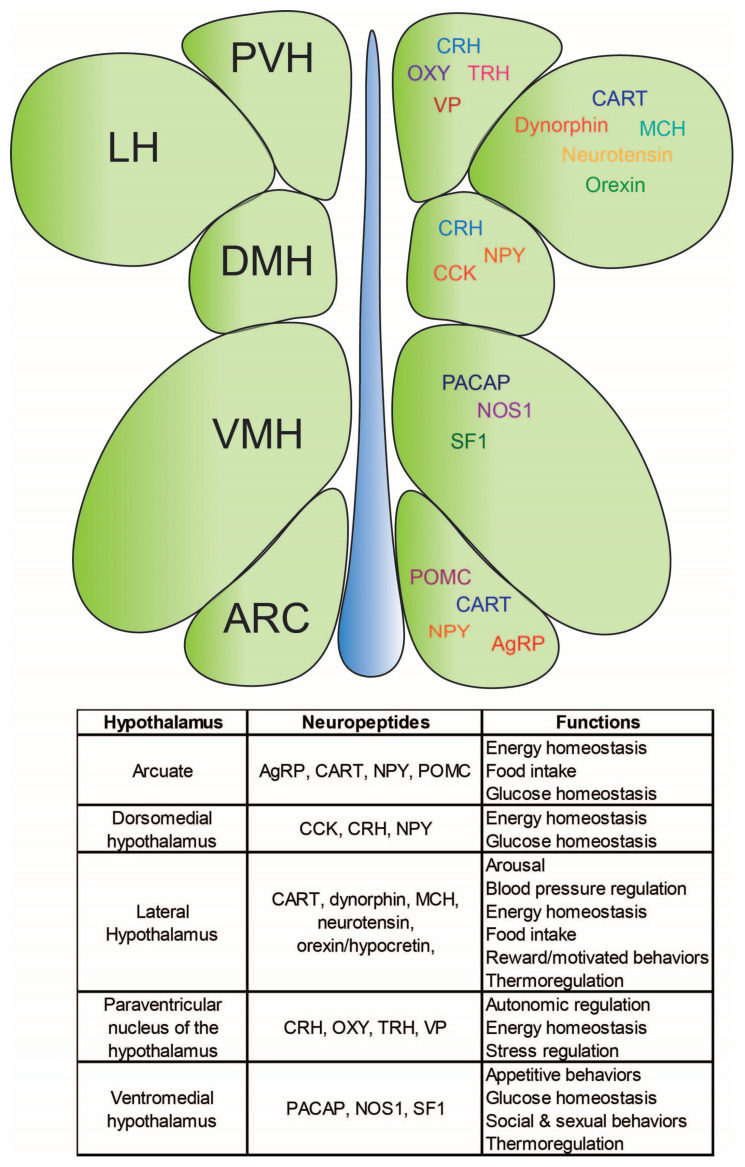
A representation of hypothalamic subnuclei as well as the distribution of neuropeptides expressed in various hypothalamic subnuclei.

The functions of the hypothalamus are incredibly diverse with many of its nuclei dedicated to regulating homeostatic processes (Figure 3). The neuronal composition of the hypothalamus underlies its plethora of functions and, as such, each hypothalamic subnucleus is heterogeneous with regard to its neuron subtypes. The sagittal hypothalamus can be divided into three distinct regions: the periventricular, medial, and lateral areas; and the periventricular area so named given its proximity to the third ventricle.

### 3.1. The Arcuate Nucleus: Integration Site for Body Energy Homeostasis

In the periventricular area, the arcuate nucleus (ARC) is a circumventricular organ that serves as an interface between endocrine and neural systems and is situated dorsally to the median eminence with some of its neurons descending directly into the median eminence [20]. The composition of the ARC is relatively heterogeneous with many of the neurons co-expressing both neurotransmitters and peptides. Pro-opiomelanocortin (POMC) neurons are found in the ARC and have diverse biological effects when activated. POMC is an anorectic precursor peptide that, when cleaved, produces several endocrine products such as α-melanocyte-stimulating hormone (MSH), β-endorphin, and adrenocorticotropic hormone (ACTH). Agouti-related peptide (AgRP) is an orexigenic hormone also found in the ARC. AgRP is typically co-expressed with neuropeptide Y (NPY) in ARC neurons to produce peptide products that stimulate food intake and decrease energy expenditure [21,22]. Previous research heavily implicates these neuropeptides in the pathophysiology of obesity. For example, while mutations in POMC are relatively rare (approximately 1 in 10,000), clinical cases of obesity have been associated with genetic aberrations of this hypothalamic neuropeptide [23,24,25,26]. Interestingly, mouse models have recapitulated this phenotype, with knockout of *Pomc* producing morbidly obese, hyperphagic mice [27].

### 3.2. The Lateral Hypothalamus: The “Hunger Center”

The lateral hypothalamus (LH) and paraventricular nucleus of the hypothalamus (PVH) are other key components that regulate body energy balance and receive anorectic and orexigenic signals stemming from the ARC. The LH historically was considered to be the “hunger” center of the brain as electrolytic lesions of this structure led to emaciated or starving rats [28]. This notion was largely dismissed after a preponderance of data demonstrated that a large network of hypothalamic and hindbrain nuclei contributed to hunger and that the LH was not the lone impetus of hunger [29]. Still, several laboratories have shown that stimulation of the LH elicits robust and ravenous bouts of feeding [30]. Pharmacologically, it has been demonstrated that injections of glutamate receptor agonists elicit feeding [31] while GABA receptor agonists inhibit feeding when directly infused into the LH [32]. Interestingly, rodents and humans alike will self-stimulate the LH inexorably to produce hedonic or pleasurable sensations suggesting that the LH may not be so much a hunger center as it is a center for motivation/reward [33]. Given that the LH also contains a dizzying array of peptides/neurohormones, its role in many biological processes such as regulating sleep–wake cycles, stress, energy balance and reward [34] suggest its importance in the context of homeostasis in general. There is also evidence that the LH is necessary to learn cue–reward associations [35,36] and has been postulated to be the intermediary between reward-related areas such as the nucleus accumbens to homeostatic ones like the ARC [37]. The LH is incredibly heterogeneous with regard to its neuropeptide populations with orexin/hypocretin, melanin-concentrating hormone (MCH), dynorphin, cocaine- and amphetamine-regulated transcript (CART), and neurotensin being some of the peptides manufactured there [34,38,39,40,41,42,43,44].

### 3.3. The Paraventricular Nucleus of the Hypothalamus: The Central Site for Homeostatic and Autonomic Function

The PVH is located adjacent to the third ventricle and positioned dorsally to the ARC. It also contains a plethora of cell types, such as parvocellular, magnocellular, and long-projecting neurons [45]. The PVH is intimately connected with the pituitary gland and releases humoral factors to activate the hypothalamic–pituitary–adrenal (HPA) and hypothalamic–pituitary–thyroid axes [46]. Parvocellular neurons of the PVH manufacture thyroid-releasing hormone (TRH) and corticotrophin-releasing hormone (CRH) to exert downstream effects on the anterior lobe of the pituitary gland [46]. Magnocellular neurons produce and secrete vasopressin and oxytocin into the posterior lobe of the pituitary. Vasopressin is a vasoconstrictor that is important for maintaining body fluid balance while oxytocin is a key humoral factor in the body’s milk let-down response [46]. Long-projecting PVH neurons express melanocortin receptors (MCRs) and also manufacture oxytocin, both of which have been implicated in body energy homeostasis [47,48]. These neurons send projections to the hindbrain and are the postsynaptic targets of AgRP neurons from the ARC, thereby making these neurons key players in energy balance [45,47,48]. The functions of the PVH include stress regulation [49], autonomic regulation [50], and metabolic function [51] and are integral to the melanocortin pathway. An extensive body of research illustrates the immense role that MCRs play in obesity. Given the high expression of MCRs in the PVH, this underlies the significant role that the PVH plays in body energy homeostasis.

### 3.4. The Ventromedial Hypothalamus: The “Satiety Center”

The ventromedial hypothalamus (VMH), like many other subregions of the hypothalamus, is involved in regulating glucose, appetitive behaviors, thermoregulation, as well as social and sexual behaviors [52]. Most of the neurons in the VMH are glutamatergic but there are other VMH markers that distinguish it from other hypothalamic areas such as pituitary adenylate cyclase-activating peptide (PACAP) [53], nitric oxide synthase 1 (NOS1) [54], as well as a unique marker known as steroidogenic factor-1 (SF1) [55]. In landmark studies performed by Hetherington and Ranson in the 1940s, electrolytic lesions of the VMH resulted in hyperphagia and rapid onset of obesity in rat models [56]. Based on these data, this hypothalamic area was coined the “satiety” center, although this particular designation may not be the most appropriate as there are other regions of the central nervous system (CNS) important in signaling satiety. With the advent of modern neuroscience, it was discovered that knockout of SF1 as well as SF1-speciifc knockout of the leptin receptor LepR produce a similar phenotype to electrolytic lesions of the VMH [57,58]. Other studies have shown that the VMH is a key regulator of glucose levels as stimulation of the VMH increases glucose production and uptake [59,60,61].

## 4. The Central Melanocortin System

Melanocortins are a particular category of neuropeptides that are integral to the central control of energy balance and feeding. These neuropeptides are products of ARC POMC and AgRP neurons and include the melanocortins, α-MSH and AgRP, respectively. These neuropeptides once manufactured by ARC POMC and AgRP neurons are shuttled to distal parts of the hypothalamus such as the PVH and LH to exert either anorectic (α-MSH) or orexigenic (AgRP) effects. α-MSH and AgRP bind to melanocortin receptors MC3R and MC4R, which are abundantly expressed in the hypothalamus and in hindbrain areas such as the lateral parabrachial nucleus (LPBN), area postrema (AP), and in the nucleus of the solitary tract (NTS) [62].

An anorectic hormone produced by white adipose tissue (WAT) adipocytes known as leptin causes the release of α-MSH from POMC neurons. α-MSH will then bind to MC3R and MC4R on PVH neurons to exert anorectic effects on food intake [62,63]. POMC mutations in both transgenic animal models and in humans result in hyperphagia, impairments in glucose homeostasis, decreased energy expenditure and early-onset obesity [23,27,64,65,66,67]. Interestingly, mutations in neural enhancers of *Pomc* (nPE1 and nPE2) lead to obesity in animal models [68], indicating that regulatory elements of POMC also exert profound effects on body energy homeostasis.

AgRP is another key peptide in the melanocortin pathway that functions as a receptor antagonist to melanocortin receptors MC3R and MC4R in the forebrain and hindbrain areas [62]. Ghrelin, an orexigenic hormone made by the stomach, binds to its receptors located on ARC AgRP neurons. Activation of these neuropeptidergic neurons through ghrelin binding leads to the release of AgRP, which then inhibits melanocortin receptors in the PVH, LH, bed nucleus of the stria terminals, and elsewhere to stimulate feeding behavior under fasted conditions [62]. Under sated conditions, leptin suppresses the activity of ARC AgRP neurons, which serves to eliminate conflicting signals to MC3R- and MC4R-containing nuclei that may arise from leptin-activation of POMC neurons [69]. Previous studies reveal that ARC-specific AgRP ablation or optogenetic inhibition of ARC AgRP neurons leads to significant weight loss and starvation in mouse models [70,71]. Overexpression in transgenic mouse models leads to hyperphagia and obesity [72], while activation or optogenetic stimulation of ARC AgRP neurons elicits spontaneous food intake in *ad libitum*-fed mice [71,73,74].

## 5. The Complex Issue of Obesity and the Impact of Ultra-Processed Foods

The prevalence of obesity in the United States has risen at an exponential rate with projected obesity and severe obesity rates to reach 50% and 25%, respectively by 2030 [75]. These alarming statistics have led to a more concerted effort by biomedical researchers to uncover the physiological, neurobiological, genetic, and epigenetic mechanisms responsible for this precipitous rise with the National Institutes of Health dedicating approximately USD 1.5 billion on obesity-related research alone for 2023 [76].

There are numerous factors that contribute to obesity pathophysiology, not least of which is genetics. To date, there are at least 27 transgenic rat and mouse models that are being used to identify the genetic underpinnings of obesity [77]. One considerable limitation of these animal models is that human clinical cases of obesity are typically polygenic with each gene only contributing a fraction of obesity risk to patients [77]. Thus, studying these genes in isolation will only provide so much insight into the etiology of obesity.

While genetics confers significant risk to obesity pathology, many other factors ultimately coalesce to precipitate the development of obesity. Sedentary lifestyles, aging, as well as dietary habits have profound impacts on our resulting phenotypes. In particular, excessive calorie intake through increased exposure to ultra-processed foods has drastically altered metabolic phenotypes, so much so that obesity is now more commonplace than ever before [78].

The allostatic shift in response capacity, whereby obese individuals are consuming in excess of metabolic need, may not have been the result of any idiopathic changes to our genome but may have been in response to modern-day eating habits, whereby ultra-processed foods (UPFs) feature largely in Westernized diets. It has been suggested that UPFs and changes to food manufacturing have fundamentally altered the diversity of our gut microbiomes [6,79]. Current studies report a substantial increase in UPF intake from 53.5 to 57% kcal (2001–2002 and 2017–2018, respectively) while consumption of minimally processed or whole foods decreased from 32.7 to 27.4% kcal over that same time period [80]. Children, in particular, have increased their dietary intake of UPFs (mean age 10.7) substantially from 61.4 to 67% during 1999–2018, while minimally processed food intake decreased from 28.8% to 23.5% [81]. Given the robust association between childhood and adult obesity, these data suggest that UPFs may be a significant factor in the developmental trajectory of obesity.

While UPFs are an abundantly accessible food source to many [82], they often lack nutrients vital to maintaining gut microbiome integrity. Food manufacturing renders food deficient in nutrients via its processing. UPFs contain an increased amount of more refined sugars and starches, otherwise known as “acellular” carbohydrates [6]. These acellular carbohydrates are more rapidly digested and thus nutrients from this form of processing are not efficiently absorbed by the gut microbiome [83,84]. This, in turn, creates a formidable breeding ground under which inflammatory conditions flourish. The risk for obesity increases substantially commensurate with UPF intake [85,86,87], indicating the potentially obesogenic effects of UPF consumption. UPFs are clearly implicated in obesity etiology, but mechanistically, how do UPFs fundamentally alter our physiologies to such an extent that permanently reversing obese states presumably becomes an insurmountable task?

This is a difficult question to answer; however, animal studies have modeled different aspects of UPFs by using either high-fat, high-sugar, or a combination of both high-fat and -sugar diets to determine their effects on hypothalamic function. These experiments revealed that exposure to a high-fat/high-sugar diet during adulthood or perinatally changes the expression of pro- and anti-inflammatory cytokines and leads to gliosis in the ARC, the effects of which are not fully reversible upon switching to a UPF-free diet [88,89]. High-fat/high-sugar-diet-fed mice have decreased fMRI signal in the ARC, lateral hypothalamus, VMH, and DMH [90] while LH GABAergic activity is suppressed after access to a high-fat diet under free-choice conditions [91]. Collectively, these data indicate that diets that mimic the nutrient profile of UPFs produce fundamental changes to the hypothalamus that may not be reversible. Another way in which high-fat/high-sugar diets may alter our physiologies is through epigenetic mechanisms.

## 6. How Environmental Factors Fundamentally Alter Our Epigenetic Landscape

### 6.1. Why Have Obesity Rates Risen Despite a Stable Genome?

Genetic variants that underlie obese pathophysiology account for a fraction of this metabolic phenotype and are not considered significant risk factors for the development of obesity [8,92,93,94]. In fact, the concordance rate of BMI between monozygotic twins is between 40 and 70%, indicating that other factors beyond genetics contribute to body weight phenotype [95]. How is it then that obesity rates have tripled in the United States over the last 40 years without concurrent changes to our genome? The paucity of monogenic forms of obesity indicates that polygenic is the more commonplace of the two, with rare variants of major candidate genes only accounting for approximately 5–10% of obesity cases [93]. In polygenic forms of obesity, each genetic aberration only makes a minor contribution to obesity, and thus, the combined effect of many gene variants is likely responsible for obese pathology [8].

Exposure to UPFs, high-caloric diets, high-fat diets, sedentary lifestyles as well as chronic stress are contributing factors to the obesity equation, but they in and of themselves do not exert direct changes to gene function. What mechanisms then are driving these changes in gene activity? It has been speculated that alterations in gene activity are mediated by the epigenome, which serves as the interface that regulates interactions between external (i.e., environment) and internal (i.e., genes) factors [96,97].

### 6.2. Epigenetics: The Interface Between Genes and Environment

Epigenetics has been conceptualized as the “molecular” bridge between our genes and our constantly evolving phenotype [98] and has historically been used as an explanation for developmental phenomena such as X-chromosome inactivation, genomic imprinting, and determination of cell phenotype [99]. Within the last three decades, however, epigenetic mechanisms have been shown to exert their influence long past early developmental stages and have emerged as a “mechanistic interface” between genes and the environment during adulthood [99]. Epigenetics has been implicated in diverse central processes such as addiction, stress, learning and memory, and cognition, and may underlie the psychopathology of mental health disorders such as depression and anxiety [100,101]. Epigenetics has provided a more parsimonious account of how gene expression reflexively changes in response to life experiences and how these alterations can result in maladaptive states such as addiction, depression, anxiety, and obesity, particularly in genetically predisposed individuals.

The term epigenetics, loosely defined, is simply heritable changes to gene expression without changes to its nucleotide sequence. Epigenetic mechanisms include DNA methylation as well as structural changes to chromatin via histone modifications [99]. Methylation of DNA has been well characterized and is the process through which a methyl group is added to the fifth carbon of the cytosine pyrimidine ring within CpG dinucleotides by DNA methyltransferases (DNMTs) during replication and de novo methylation [7,102]. Alternatively, demethylation can also occur through ten-eleven translocation (TET) enzymes by oxidizing 5-methylcytosines [103]. Post-translational modifications to histone proteins are a different category of epigenetic mechanisms that involve structural changes to the histone/DNA complex. This type of structural remodeling requires histone modifications in the form of acetylation, phosphorylation, ubiquitination, methylation [104], demethylation [105], isomerization [106], acylation [107], as well as succinylation [108]. Histone lactylation is a recently discovered histone modification whereby lactate attaches to lysine residues located on histone proteins, the results of which increase gene transcription from chromatin [109]. Succinylation is another posttranslational modification in which a succinyl group is added to the ε-amine of a lysine residue and can occur on histone proteins to increase gene accessibility or on chromatin, which is positively correlated with gene expression [108]. Epigenetic modifications also include RNA-dependent mechanisms such as siRNA, long non-coding (lnc) RNA, as well as miRNA. This latter category of epigenetic modifications influences transcription via DNA methylation (siRNA, miRNA), histone modification (siRNA, miRNA), or chromatin restructuring (lncRNA) [110].

### 6.3. Early Life Exposure to Obesogenic Diets and Epigenetic Programming

A preponderance of data in animal models indicates that epigenetic mechanisms are altered by early exposure to obesogenic diets and may lead to an increased propensity to develop obesity later in life. Changes in epigenetic function as a result of early developmental exposure to obesogenic diets may be key to understanding why obesity is such a difficult state to overcome and why relapses in obesity are so common. Previous work has identified differentially methylated regions in the hypothalamus after exposure to high-calorie or normal-chow diets perinatally [111]. Additionally, several studies have shown that hypermethylation of the *Pomc* gene is seen in mouse models in which dams are exposed to a high-fat diet [112,113], similar to what has been observed in clinical cases of obesity [114,115]. Methyl-CpG binding protein 2 (MeCP2) is an epigenetic factor that binds to highly methylated regions of DNA in order to repress transcription [116,117]. Previously published data show that MeCP2 may play a role in early developmental processes involved in body energy homeostasis as decreased MeCP2 protein expression in the ARC is observed in a sexually dimorphic manner after perinatal exposure to a high-fat diet [118]. Interestingly, hypomethylation of the ARC *Pomc* gene is evident in C57/BL6 male mice exposed to a low-protein diet *in utero* and then weaned onto a high-fat diet [119,120]. Hypomethylation of ARC *Pomc* is also seen in mice offspring maintained gestationally and then weaned onto a high-fat/sugar diet [113]. Other data show that loss of POMC-specific MeCP2 expression early in development [121] leads to increased body weight, elevated plasma leptin levels, increased DNA methylation of the *Pomc* promoter, and increased conditioned place preference for a high-fat diet [122,123]. Collectively, these data suggest that early perinatal exposure to obesogenic diets can result in profound changes to epigenetic programming that may eventually predispose organisms towards obesity.

### 6.4. Diet-Induced Epigenetic Changes in Adulthood

Expression of epigenetic factors is also dynamically regulated as a function of diet during more mature periods of development such as adulthood or adolescence. Histone deacetylases (HDACs) are enzymes that remove acetyl groups from lysine residues, a process associated with gene repression [124,125]. Medial hypothalamic expression of HDACs 5 and 8 are significantly increased after 4 weeks of a high-fat diet while sixteen hours of fasting increases HDACs 3 and 4 levels and decreases HDACs 10 and 11 levels in the medial hypothalamus of adult mice [126]. While medial hypothalamic HDAC 11 expression is decreased in fasted mice, knocking out this class-IV HDAC has been associated with resistance to diet-induced obesity and leads to improvements in glucose tolerance and insulin sensitivity [127]. Inhibition of HDAC 6 through peripheral administration of tubastatin A reduces obesity and restores leptin sensitivity in diet-induced obese mice, suggesting that epigenetic mechanisms may exert profound effects on body energy homeostasis [128]. Another class of HDACs known as sirtuin1 (SIRT1) influences chromatin state and is upregulated in the hypothalami of pups born from dams fed a high-fat diet during gestation [129]. When this particular HDAC is knocked out in ARC POMC neurons, mice become hypersensitive to diet-induced obesity (DIO) [57]. SIRT1 inhibitors injected centrally inhibit ARC AgRP neuron activity and decrease inhibition of POMC neurons, leading to suppression of food intake during the dark cycle and after treatment with the orexigenic hormone, ghrelin [130]. Knockout of *Sirtuin3* in mice also produces obesity and metabolic syndrome particularly when challenged with obesogenic diets [131].

HDACs are not the only epigenetic modification that impacts body energy homeostasis. Methylation and demethylation processes have also been implicated in obese pathophysiology. Rapps et al. [132] demonstrate that exposure to DIO downregulates *Kdm4d* mRNA, which increases methylation of H3K9me2 on AgRP’s promoter region and transcriptionally silences AgRP. Conversely, calorically restricting rats after DIO produces demethylation of H3K9 on the AgRP promoter with a concomitant increase in AgRP transcription [132]. Exposure to a high-fat diet during adolescent periods of development is associated with a decrease in DNA 5-hmC, an intermediary of DNA demethylation, in the ventromedial hypothalamus of male mice and remains decreased even after cessation of HFD [133].

lncRNAs have been heavily implicated in epigenetic modification, adipose tissue biology, and energy metabolism. Of particular interest is an lncRNA known as *AK044061*, which is upregulated in the hypothalamus after exposure to DIO and when expressed in the ARC produces obesity in mice. Alternatively, DIO is prevented by AgRP-specific knockdown of *AK044061* [134]. NF-κB is a transcription factor implicated in leptin signaling [135] and *Pomc* transcription [136] and has been shown to alter chromatin structure [137]. *AK044061* has been shown to influence NF-κB transcriptional activity in order to exert downstream effects on body weight homeostasis [134].

Others have shown that newly discovered epigenetic modifications, such as histone lactylation, may play an important role in the pathophysiology of obesity. For example, Chen et al. [138] show that interruptions in histone lactylation, a process whereby histones are altered by the addition of lactyl groups to lysine residues, produce bidirectional changes to body weight. Fam172a is a protein that regulates histone lactylation. Specifically, knockdown of Fam172a in POMC neurons protects against DIO while overexpression of Fam172a in POMC neurons produces an obese phenotype in mice fed a normal-chow diet [138], presumably by influencing glycolytic processes which lead to histone lactylation of α-MSH [138].

### 6.5. The Persistence of Epigenetic Memory in Obesity

The aforementioned data demonstrate that central epigenetic processes such as methylation, demethylation, acetylation, lactylation, etc., are influenced by environmental stimuli such as HFD and are dynamically regulated over time. At its core, obesity is a relatively enduring condition. A meta-analysis revealed that more than 75% of weight loss is regained after 5 years [139,140]. The cause of this allostatic shift in body energy homeostasis, whereby the body is incapable of maintaining permanent weight loss, has not yet been elucidated.

**Figure 4:**
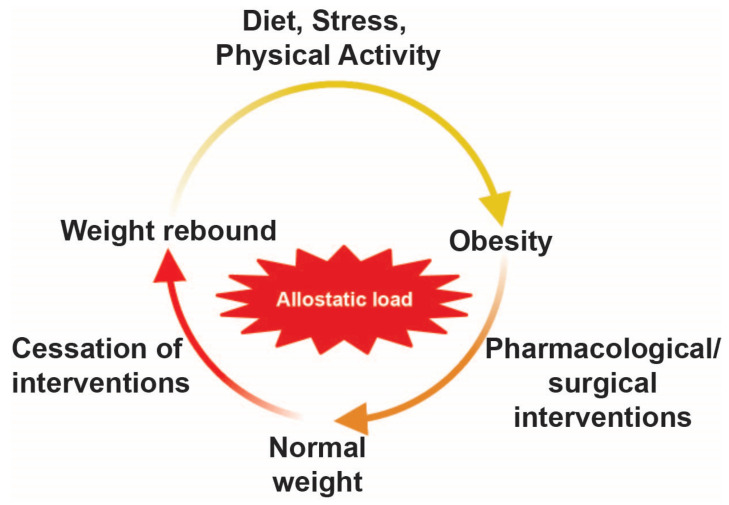
External variables (i.e., high-caloric diets, decreased physical activity, stress) increase propensity to develop obesity. Obesity can be temporarily reversed by pharmacological/surgical interventions but can be reversed over time after cessation of treatment, presumably through the result of increased allostatic load.

The nature of this allostatic shift is such that surgical or pharmacological interventions can sometimes be ineffectual at reversing obesity. An example of this is evident in bariatric surgery patients in which obese individuals either undergo a Roux-en-Y gastric bypass or a sleeve gastrectomy. These metabolic surgeries decrease stomach capacity by physical removal of the stomach (as is the case with sleeve gastrectomy) or by creating a small pouch of the stomach and connecting it with the small intestine (as is the case with Roux-en-Y gastric bypass). While the treatment initially appears successful, weight regain is commonplace in this population, with some reports indicating anywhere from 23 to 76% of patients seeing significant weight gain 1–6 years after surgery [10,11,12,141,142]. This similar phenomenon is seen after the cessation of popular weight loss drugs, Wegovy and Ozempic. These pharmacotherapies mimic the actions of glucagon-like peptide 1 (GLP1), a hormone that slows down the rate of gastric emptying thereby attenuating hunger signals. Discontinuing treatment of these GLP1 receptor agonists leads to weight rebound in which individuals recover lost weight (Figure 4). It is possible that weight rebound is the result of adipose tissue possessing a metabolic memory [143], a process whereby adipose tissue cells “retain a metabolic memory…conferred by epigenetic changes”.

To our knowledge, data demonstrating a link between this phenomenon of weight rebound and neuroepigenetics have not yet been established. However, recent data suggest that the “epigenetic memory” [144] for obesity may be the result of epigenetic modifications to adipocytes. Exposure to an HFD perinatally induces metabolic changes in adipose tissue. Offspring of dams given an HFD during lactation have attenuated brown adipose tissue (BAT) thermogenesis, a form of adipose tissue that is metabolically active relative to white adipose tissue (WAT), whose primary function is to store fat [144]. Thermogenic genes also undergo epigenetic modifications. For example, histone methylation of genes necessary for activation of Uncoupling Protein-1 (UCP1) has been shown to lead to the browning of WAT, a more metabolically active form of fat tissue [145]. Additionally, Parathyroid Hormone-Related Protein (PTHrP), a protein implicated in adipogenesis, activates the cAMP/PKA pathway, leading to the phosphorylation of a number of proteins including SIRT1, a class-III HDAC that deacetylates Peroxisome Proliferator-Activated Receptor Gamma Coactivator 1-alpha (PGC1α), a protein that influences many metabolic pathways [146]. Recently, it was shown that obesogenic diets induce epigenetic changes at thousands of loci in adipocytes. This finding was observed in animal models and in human clinical cases of obesity and, strikingly, this altered epigenetic expression was evident in mice that were switched from an HFD to normal chow [143]. These data suggest that exposure to obesogenic diets is sufficient to impart a long-lasting “epigenetic memory” [143] in adipose tissue even after reverting back to a normal body weight, the results of which may account for shifting response capacities that prevent the rolling back of the proverbial clock (see Section 2). It is not clear at what point these response capacities transform into a more fixed state, but Liu et al. demonstrate that gestational exposure to succinate, which enhances fetal BAT development through succinylation of the *Ppargc1α* promoter, protects against HFD-induced obesity [147]. These data indicate that intervention in the form of succinate during sensitive periods of development may make for a more dynamic system whereby epigenetic mechanisms such as succinylation can be more responsive against perturbations such as excess energy balance.

### 6.6. Bridging Animal Research and Human Clinical Studies

While human clinical work has its limitations, it has nonetheless provided some insight into putative epigenetic mechanisms underlying the pathogenesis of obesity. For example, exposing human participants to a high-fat overfeeding protocol altered methylation patterns in skeletal muscle and adipose tissue [148,149,150,151]. These obesogenic diets have pronounced and long-lasting effects on methylation status as these changes are not easily overcome by reverting back to a “normal” diet. DNA methylation of thousands of CpG sites was strongly associated with BMI in adipose tissue [152]. In obese women with gastric bypass surgery, DNA methylation from fat cells was compared with weight-matched women. Using fat cells, it was shown that over 8000 CpG sites had differential methylated DNA sites in post-obese women with gastric bypass surgery, with 27% associated with adipogenesis relative to never-obese controls [153].

Interestingly, clinical data have also reported altered expression of HDACs in adipose tissue. Changes to HDAC expression are also evident in clinical cases of obesity as HDAC 5 is decreased in subcutaneous adipose tissue while HDAC 4 is decreased in visceral adipose tissue of obese adult women [154]. While more work is necessary, it is evident that epigenetic mechanisms are dynamically regulated by obesogenic diets and may in turn impart lasting effects on metabolic phenotype that perhaps are indicative of the allostatic shift associated with obese pathology.

Changes to the human epigenome are not restricted to adipose tissue but are also evident in central mechanisms as well. For example, a strong association was observed between epigenetic variation and obesity risk, with data demonstrating increased DNA methylation of *POMC* in adolescents and adults with obesity [109]. POMC-expressing neurons differentiated from human embryonic stem cells were shown to be hypermethylated with a concomitant reduction in *POMC* gene expression [155]. This increased methylation was observed in a variable methylated region of the *POMC* gene. These clinical data suggest that central epigenetic mechanisms are influenced by obesogenic diets and may contribute to obese pathology.

Alterations in methylation status are mediated by DNMTs and mutations in the genes that encode for these enzymes may be another candidate epigenetic mechanism underlying the etiology of obesity. For example, mutations in DNMT3a lead to the development of a rare disorder known as Tatton–Brown–Rahman Syndrome (TBRS), which is marked by symptoms of intellectual disability, height and head circumference overgrowth, and obesity [156,157]. A transgenic mouse model of TBRS, in which DNMT3a was constitutively knocked out, recapitulated the obese phenotype and hyperphagia while exposed to either a regular or high-fat diet [158,159].

These data collectively suggest a significant link between epigenetic mechanisms and obesity pathogenesis. The caveat to these clinical findings, however, is the paucity of data linking changes to the epigenome at the central level, experiments that may be more amenable to animal models. Data from transgenic animal models have brought significant insight into neuroepigenetic mechanisms affected by environmental factors such as obesogenic diet. Bridging the conceptual gap between human clinical studies and animal research is the next critical step to identifying neuroepigenetic factors that underlie the pathophysiology of obesity. The effects of UPFs on our epigenomes are insufficient at this time and more work is needed to determine the cumulative effects UPFs have on body energy regulatory mechanisms. The insights provided by such research could be key to unlocking the mystery of the precipitous rise in and enduring effects of obesity.

## 7. Conclusions

Taken together, the aforementioned data indicate that environmental factors such as a high-fat diet, which models aspects of UPFs, can have profound impacts on our epigenome. UPFs and other environmental factors (i.e., stress, sedentary behavior, etc.) produce an increased allostatic load that exerts profound changes to the epigenome that result in a fixed state of obesity. Thus, obesity becomes the “new normal” for the organism. Simply put, the organism adapts to high-calorie, high-fat diets and preserves this new set point by maintaining excessive calorie intake, to the detriment of the organism. The organism does not necessarily recognize this new state as maladaptive or injurious but instead defends its shifted higher set point. This may be why overcoming obesity is daunting and why it is such a persistent, life-long condition for some. According to Hinte et al., exposure to obesogenic diets alters epigenetic memory in adipose tissue in such a way as to render these tissues resistant to weight loss diets [143]. Thus, individuals’ weight loss efforts are undermined by the retention of epigenetic memory [143]. Collectively, these data indicate that aberrant metabolic processes are difficult to reverse unless done so under the prolonged influence of pharmacological treatments and that finding the source of epigenetic memories, whether that be in adipose tissue or in central mechanisms, may provide insight into the most effective ways to reverse these types of persistent molecular changes.

While there are no clear-cut answers to mitigating the current obesity crisis, what remains evident is that the treatment of obesity, particularly in individuals who are co-morbid for other disease states and mental health disorders, is of paramount importance. Reversing obesity can lead to vast improvements not only in physical health but also in quality of life, and given the incredible diversity of factors underlying obesity, perhaps a tailor-made approach to its treatment may be more beneficial than a one-size-fits-all approach. Our current diets are exerting a profound influence on our epigenome so much so that it is creating substantial shifts to our physiologies, maintaining an untenable condition with deleterious consequences. More work on the effects of UPFs and obesogenic diets on our epigenomes is necessary to fully appreciate how our physiologies are impacted by these complex interactions. Harnessing the power of animal models will bring significant insight into how obesogenic diets and UPFs affect epigenomes and fundamentally alter central processes in order to accommodate our ever-shifting set points. Understanding the effects of diet on our epigenomes may inform novel therapeutic strategies but importantly may strongly discourage the consumption of UPFs or other obesogenic foods in Westernized societies, which may ultimately lead to the amelioration of obesity.

## Abbreviations

The following abbreviations are used in this manuscript:

AP	Area postrema
ARC	Arcuate nucleus of the hypothalamus
ACTH	Adrenocorticotropic hormone
AgRP	Agouti-related peptide
BMI	Body Mass Index
CART	Cocaine- and amphetamine-regulated transcript
CDC	Centers for Disease Control
CNS	Central nervous system
DIO	Diet-induced obesity
DNMT	DNA methyltransferase
GLP1	Glucagon-like peptide 1
HDAC	Histone deacetylase
HPA	Hypothalamic–pituitary–adrenal
LH	Lateral hypothalamus
LPBN	Lateral parabrachial nucleus
MCH	Melanin-concentrating hormone
MeCP2	Methyl-CpG binding protein 2
MSH	Melanocyte-stimulating hormone
MCR	Melanocortin receptor
nPE1/2	Neural POMC enhancer 1/2
NPY	Neuropeptide Y
NTS	Nucleus of the solitary tract
PACAP	Pituitary adenylate cyclase-activating peptide
PGC1α	Peroxisome Proliferator-Activated Receptor Gamma Coactivator 1-alpha
POMC	Pro-opiomelanocortin
PTHrP	Parathyroid Hormone
PVH	Paraventricular nucleus of the hypothalamus
SF1	Steroidogenic factor-1
SIRT1	Sirtuin 1
TET	Ten-eleven translocation
TBRS	Tatton–Brown–Rahman Syndrome
TRH	Thyroid-releasing hormone
UCP1	Uncoupling Protein 1
UPF	Ultra-processed foods
VMH	Ventromedial hypothalamus
WAT	White adipose tissue
WHO	World Health Organization
